# The effect of carbon monoxide releasing molecule-2 (CORM-2) on healing of ischemic colon anastomosis in rats

**DOI:** 10.3906/sag-1902-43

**Published:** 2021-08-30

**Authors:** Ali Kemal KAYAPINAR, Metin ERCAN, Kadir Koray BAŞ, Seda YAMAK, Uğur ERÇİN, Mehmet Akif TÜRKOĞLU, Erdal Birol BOSTANCI

**Affiliations:** 1 Department of General Surgery, Tepecik Training and Research Hospital, University of Medical Sciences, İzmir Turkey2; 2 Department of Gastroenterology Surgery, Faculty of Medicine, Sakarya University, Sakarya Turkey; 3 Department of General Surgery, Bozyaka Training and Research Hospital, University of Medical Sciences, İzmir Turkey; 4 Department of Pathology, Ankara City Hospital, University of Medical Sciences, Ankara Turkey; 5 Department of Medical Biochemistry, Faculty of Medicine, Ufuk University, Ankara Turkey; 6 Department of General Surgery, Faculty of Medicine, Gazi University, Ankara Turkey; 7 Department of Gastroenterology Surgery, Ankara City Hospital, University of Medical Sciences, Ankara Turkey

**Keywords:** Anastomotic leak, angiogenesis, necrosis, ischemia, carbon monoxide releasing molecule-2 (CORM-2)

## Abstract

**Background/aim:**

Ischemia on the colon wall negatively affects healing of anastomosis. We were aimed to evaluate the effects of carbon monoxide releasing molecule-2 (CORM-2) on the healing of anastomosis in a rat model of the ischemic colon.

**Materials and methods:**

In this prospective study a total of 60 rats were randomly divided into three groups as colon transection and end-to-end anastomosis (Group I), colon transection, and end-to-end anastomosis following the induction of ischemia (Group II), and colon transection and end-to-end anastomosis following the induction of ischemia and treated with daily intraperitoneal administration of CORM-2 (Group III). Each group was also divided into two equal subgroups as postoperative 3rd and 7th day. Postoperative healing of anastomoses was evaluated by anastomosis burst pressure (ABP), tissue biomarkers including hydroxyproline (HP), malondialdehyde (MDA), glutathione (GSH), and histopathological findings.

**Results:**

In the ischemic group treated with CORM-2, lower MDA and higher HP levels were observed in comparison to the untreated ischemic group on the 3rdday. GSH and HP levels were higher and MDA levels was lower in the ischemic rats treated with CORM-2 than in the ischemic untreated rats on the 7th day. In the ischemic group treated with CORM-2, the mucosal epithelial score decreased and the neoangiogenesis score increased compared to the untreated rats on the 7th day.

**Conclusion:**

In ischemic colon anastomosis, reduces cell destruction by suppressing the oxidative reaction, and strengthening the antioxidative mechanisms of the cells. It also increases collagen formation, epithelial development, and neoangiogenesis.

## 1. Introduction

Gastrointestinal (GI) anastomosis procedure is frequently performed both as emergency cases and elective surgery. Bacteria-laden intestinal content may spread into the intraabdominal zone thus causing complications such as intra-abdominal abscess, sepsis, and multiple organ failure that may lead to death unless the anastomosis is sufficient [1,2].

The strategic decision of surgical anastomosis technique dependes on many factors such as comorbid diseases, anastomosis tension, peritonitis, sepsis, and ischemia. The latter is one of the important factors affecting the healing process [3]. Ischemia in the anastomosis may occur due to arteriosclerotic vascular disease, advanced age, inflammatory vascular disease, vasoconstrictor drug use, and vascular network damage during the surgical manipulation [4,5].

During the GI anastomosis, the initial goal is to achieve vasoconstriction for hemostasis. Following this stage, insoluble hemostatic matrix structure consisting of neutrophils, macrophages, and thrombocytes is formed in the incision area by means of inflammatory mediators [6,7].

In case of ischemia in the GI anastomosis, the inflammatory agents and radical oxygen metabolites (ROM) surge with the increase of cytokines, adhesive and vasoactive molecules during the healing process [8]. ROM modifies the pH of the environment, causing break down of the organelles and cell death [9]. In addition, the severity of inflammatory reaction disrupts the balance between synthesis and degradation of collagen by preventing collagen synthesis and leads to anastomotic failure [10].

Motterlini et al. [11] stated that CORM-2 has a vasodilator, antiischemic, and antiinflammatory effect in both in vivo and in vitro environments. In a rat models with ischemia/reperfusion damage of the small intestine, CORM-2 has been shown to reduce the damage via antioxidant, antiinflammatory, and antiapoptotic effects [12]. Ahanger et al. [13] found that CORM-2 suppresses the formation of proinflammatory adhesion molecules and increases the release of antiinflammatory cytokines, collagen production, and neovascularization in the skin full-thickness wound model.

In this study, for the first time in the literature, we aimed to evaluate the effect of CORM-2 on healing of colon anastomosis by (i) analyzing the oxidative stress through tissue malondialdehyde (MDA) and glutathione (GSH) levels, (ii) evaluating the histological necrosis rate and healing stages by histopathological examination, and (iii) determining the healing degree of anastomosis by measuring tissue hydroxyproline (HP) and anastomosis burst pressure (ABP) in experimental ischemia-induced colon anastomosis model.

## 2. Materials and methods

### 2.1. Ethical considerations and experimental design

This prospective experimental study was conducted at Ankara University, Laboratory of Experimental Animals after the approval of the Ethics Committee of Ankara University, Experimental Animals (Approval Date: 09.05.2012; no.: 2012-10-70).

**Figure 1:**
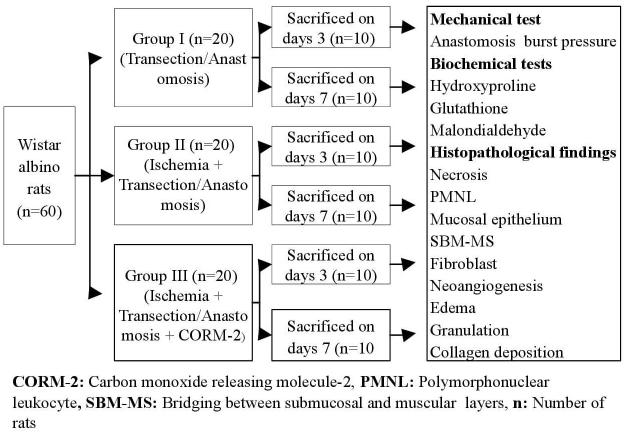
Research design. n: Number of rats; CORM-2: carbon monoxide releasing molecule-2; PMNL: polymorphonuclear leukocyte; SBM-MS: bridging between submucosal and muscular layers.

A total of 60 female Wistar albino rats weighing between 180–220 g were kept in a specialized shelter (The rats were fed with standard chow and tap water ad libitum and lived at 12:12 h light-dark cycle with room 13 temperature at 21 °C as standard animal care condition) and were healthy during the preoperative period. All rats were randomly and equally divided into Group I (n = 20), Group II (n = 20), and Group III (n = 20), and underwent surgical procedures described below. The research design is shown in Figure 1. 

### 2.2. Surgical procedure and applications 

Following anesthesia with intraperitoneal xylasine (5 mg/kg) + ketamine (50 mg/kg), the procedures described below accompanying laparotomy were performed. 

Group I (transection/anastomosis): The colon was dissected completely 3 cm proximal to the pelvic peritoneum. The abdomen was closed after end-to-end anastomosis of intestinal edges with 8 primary sutures to make a single layer by using 6/0 polypropylene. Group II (ischemia + transection/anastomosis): The incision site of the colon was determined 3 cm proximal to pelvic peritoneum. To achieve ischemia in the anastomosis line 2 cm proximal and 2 cm distal of mesenteric vessels (vasa recta) were ligated. The abdomen was closed after end-to-end anastomosis of intestinal edges with 8 primary sutures to make a single layer by using 6/0 polypropylene. Group III (ischemia + transection/anastomosis + CORM-2): the same surgical procedure was implied as in Group II. 

The operated rats were placed in seperate sections from other rats and daily wound care was performed. They were checked every 15–20 min and followed up by providing liquid and food support. 

In the postoperative period (including the day of operation), intraperitoneal saline was given to Group I (n = 20) and Group 2 (n = 20) once daily and intraperitoneal CORM-2 (dissolved in 0.75% dimethyl sulfoxide prepared with saline 5 mg/kg) was given to Group III (n = 20) until the rats were sacrificed. Each group was also divided into two equal subgroups (n = 10) according to the days of sacrification on day 3 and 7. 

All rats underwent re-laparotomy following anesthesia with intraperitoneal xylasine (5 mg/kg) + ketamine (50 mg/kg) on days 3 and 7. The abdominal incision was re-opened and the colonic anastomosis was identified. The rats were sacrificed following the resection of the colon segment containing 3 cm proximal and distal to anastomosis line. The dense omentum and bowel adhesions were not separated from the anastomosis to preserve the integrity of the anastomosis. The lumen was cleaned with saline. Subsequently, the anastomotic segment was connected to an infusion pump (Braun, Germany) on one side and to a manometer (Datex-Ohmeda Compact S/5, Finland) on the opposite side. The pressure was then raised by a NaCl 0.9% solution was infused at a constant rate of 1 mL/min. Anastomotic bursting pressure (ABP) was indicated as a sudden loss of pressure and defined as the maximum intraluminal pressure prior to leakage.

The resected segment was opened longitudinaly and divided into three equal parts after the removal of 0.5 cm proximal and distal anastomosis sections. The first piece was placed in 10% formaldehyde for histopathological examination. The other two parts were frozen at –40 ^o^C with liquid nitrogen for the measurement of HP, MDA, and GSH levels. 

### 2.3. Biochemical analyses

#### 2.3.1. Hydroxyproline (HP) analysis

After addition of 6N hydrochloric acid (HCl) onto 0.1 g of colon tissue that were weighed and transferred to the Pyrex tubes, they were hydrolyzed at 105 ^o^C for 18 h. 25 mL of samples of hydrolysates were lyophilized and dissolved in 1.2 mL of 50% (v/v) isopropyl alcohol. Chloramine T solution was added to these samples and after 10-min incubation, 1 mL Ehrlich reagent was added and the samples were incubated at 50 ^o^C for 90 min. The color was analyzed on a spectrophotometer at a wavelength of 560 nm. Under the same experimental conditions, 0.4, 0.6, 0.8, 1.2, and 1.6 µg HP standards were also analyzed and sample concentrations were calculated from the standard curve. The results were given as g HP/g tissue [14].

#### 2.3.2. Malondialdehyde (MDA) analysis

Colon tissues were homogenized in 1.15% potassium chloride (KCl) solution at a ratio of 1:10, and MDA levels were analyzed with spectrophotometry from homogenate by the thiobarbituric acid method. The results were determined as nanomole (nmol) MDA per g of tissue [15].

#### 2.3.3. Glutathione (GSH) analysis 

GSH levels were measured via the Ellman method. The tissues were homogenized with metaphosphoric acid and centrifuged at 2500 rpm. The supernatant was colored with dithionitrobenzoic acid and spectrophotometric measurement was performed. Protein levels were also analyzed using the same supernatant by the Lowry method and results were reported as micromole (µmol) GSH per mg tissue protein [16].

### 2.4. Histological analyses

The tissues for histopathological examination were fixed in 10% formalin solution. Samples were taken from anastomosis line and embedded in paraffin blocks. Sections of 5 μm thickness were prepared from the blocks, and routine hematoxylin-eosin staining was performed. Histopathological examination was blindly reviewed according to the modified Verhofstad/Ehrlich–Hunt scoring system by a single pathologist (Table 1), [17–19].

**Table 1:** Histopathological scoring system of modified Verhofstad/Ehrlich–Hunt.

Score	0	1	2	3
Necrosis	None	Little	Marked	Massive increased
PMNL	In normal count	Slight increase	Marked infiltration	Massive increased
MNL	In normal count	Slight increase	Marked infiltration	Massive increased
Edema	None	Slight increase	Marked	Massive increased
Mucosal epithelium	Normal glandular	Normal cubic	Missing cubic	None
Submucosal muscular layer	Good bridging	Moderate bridging	Weak bridging	No bridging
Granulation	None	Partial	Marked	Severe
Neoangiogenesis	None	Partial	Moderate	Marked
Fibroblast	None	Partial	Moderate	Marked
Collagen deposition	None	Partial	Moderate	Marked

PMNL: polymorphonuclear leukocyte; MNL: mononuclear leukocyte.

### 2.5. Statistical analysis

Statistical analysis was performed using the SPSS v. 22.0 software (IBM Corp., Armonk, NY, USA). Power and sample size were calculated by using G* Power version 3.1.9.2. Based on similar reference studies, partial eta- squared values were set at 0.25 for Analysis of Variance (ANOVA). Partial eta-squared values for three groups was 0.25 and α = 0.05. The number of rats to reach power level of 80% in each group was 27. Due to the possibility of some rats dying, additional rats were included into each group. Therefore, a total of 60 rats (30 for 3rd day and 30 for 7th day) were included in the study. 

According to the outcomes of this study sufficient number of sample size has been achieved for malondialdehyde measurement (main purpose of our study) as 99% for the group in which rats were sacrificed on the 3rd day (n1 = 10, n2 = 10, n3 = 10). The group in which rats were sacrificed on the 7th day, the partial eta-squared value was 0.362 with the power of 93%. Normal distribution fitness of the data was evaluated using the Shapiro–Wilk test and variance homogeneity using the Levene test. In the inter-group analysis, one-way analysis of variance (ANOVA) test was utilized for parametric tests to compare the quantitative data of more than two groups, while the Fisher’s least significant difference (LSD) and Games–Howell tests were used for post-hoc analysis. 

The Kruskal–Wallis test and Monte Carlo simulation were used to analyze nonparametric variables. The post-hoc analysis was carried out using Dunn’s test. Quantitative variables were expressed in mean ± standard deviation (SD), and median range (min-max), while categorical variables were expressed in n (%). A p value of < 0.05 was considered statistically significant with a 95% confidence interval (CI). 

## 3. Results

### 3.1. Anastomotic burst pressure (ABP)

There was no significant difference in the mean ABP values on the 3rd day among the groups (p = 0.068) (Table 2). On day 7, ABP of group I was higher than group II and III (p = 0.039 and p = 0.045, respectively). However, there was no statistically significant difference between Groups II and III (p = 0.769) (Table 3).

**Table 2:** Distribution of values of ABP, HP, GSH, and MDA on the day 3.

		ABP	HP	GSH (x103)	MDA
3rd day	Group	Median(Min-Max)	Mean ± SD	Mean ± SD	Median(Min-Max)
	I	9 (0–23)	426.30 ± 36.04	9.90 ± 1.20	86 (72–149)
	II	4 (0–27)	325.60 ± 66.49	10.70 ± 1.16	180 (109–235)
	III	15.5 (2–97)	443.44 ± 30.87	9.60 ± 0.84	79.5 (73–103)
P-value		0.068	<0.001	0.080	<0.001
Pairwise comparison	I→II	NS	<0.001	NS	0.003
	I→III	NS	0.441	NS	1
	II→III	NS	<0.001	NS	<0.001

One-way ANOVA test, Post Hoc test: Fisher’s least significant difference (LSD-Games Howell; Kruskal–Wallis test (Monte Carlo), Post Hoc test: Dunn’s test; NS: not significant; SD: standard deviation; Max: maximum; Min: minimum.

**Table 4:** Distribution of values of ABP, HP, GSH, and MDA on the day 7.

		ABP	HP	GSH (x103)	MDA
7th day	Group	Mean ± SD	Mean ± SD	Mean ± SD	Mean ± SD
	I	168.90 ± 37.35	349.70 ± 55.30	15.89 ± 2.47	64.89 ± 16.00
	II	87.90 ± 83.88	287.33 ± 75.73	12.60 ± 2.95	95.50 ± 23.17
	III	110.50 ± 58.95	343.20 ± 31.90	15.10 ± 2.38	67.00 ± 18.86
P value		0.033	0.047	0.027	0.003
Pairwise comparison	I→II	0.039	0.023	0.011	0.002
	I→III	0.045	0.798	0.518	0.817
	II→III	0.769	0.040	0.042	0.003

One-Way ANOVA test, Post Hoc test: Fisher’s least significant difference (LSD)-Games Howell; SD: standard deviation.

### 3.2. Biochemical findings

The HP levels of group I and III on 3rd and 7th days were higher than those of group II (p < 0.001, p < 0.001 for the 3rd day, respectively) (p = 0.023, p = 0.040 for the 7th day, respectively). However, there was no statistically significant difference in the HP levels of groups I and III (p = 0.441 and p = 0.798, respectively) (Tables 2 and 3). 

There was no significant difference in the GSH levels on day 3 among the groups (p = 0.080) (Table 2). However, the GSH levels in groups I and III were higher than group II on the day 7 (p = 0.011 and p = 0.042, respectively), although there was no significant difference between group I and III (p = 0.518) (Table 3).

On the 3rd and 7th days, the MDA levels were higher in group II than in groups I and III (p = 0.003 and p < 0.001 for the 3rd day, respectively) (p = 0.002 and p = 0.003 for the 7th day, respectively). However, there was no significant difference in the MDA levels of groups I and III (p = 1) (p = 0.817) (Tables 2 and 3).

### 3.3. Histopathological findings

On day 3, the necrosis rates of groups II and III were higher than those of group I (p < 0.001 and p < 0.015, respectively), although there was no statistically significant difference in the necrosis rates between groups II and III (p = 0.586) (Table 4). On day 7, the necrosis rates of group I was lower than those of group II (p < 0.001), but not significantly different from group III (p = 0.080). Although the necrosis rates of group III tended to decrease compared to group II, there was no statistically significant difference among the groups (p = 0.292) (Table 5) (Figures 2a and 2b).

**Table 4:** Histopathological findings of each groups on day 3.

3rd day	I	II	III	P value	Pairwise comparison		
	Median(Min-Max)	Median(Min-Max)	Median(Min-Max)		I→II	I→III	II→III
Necrosis	1 (0–2)	3 (2–3)	2 (2–3)	<0.001	<0.001	0.015	0.586
PMNL	2 (1–2)	2 (2–3)	3 (2–3)	0.006	0.027	0.008	1
MNL	1 (1–1)	1 (1–2)	1 (1–1)	0.315	NS	NS	NS
Edema	1 (0–2)	1 (0–2)	0.5 (0–2)	0.332	NS	NS	NS.
Mucosal epithelium	2 (1–2)	3 (2–3)	2.5 (2–3)	0.004	0.010	0.034	1
Submucosal muscular layer	2 (2–3)	3 (2–3)	3 (2–3)	0.010	0.008	0.035	1
Granulation	1 (1–3)	1 (1–2)	1 (0–2)	0.471	NS	NS	NS
Neoangio genesis	1 (1–2)	1 (1–2)	1 (1–2)	0.642	NS	NS	NS
Fibroblast	1 (1–3)	1 (1–2)	1 (1–2)	0.917	NS	NS	NS
Collagen deposition	0 (0–1)	0 (0–1)	0 (0–1)	0.509	NS	NS	NS

Kruskal–Wallis test (Monte Carlo), Post Hoc test: Dunn’s test; NS: Not significant; Max: maximum; Min: minimum

**Table 5:** Histopathological findings of each group on day 7.

7th day	I	II	III	P Value	Pairwise comparison		
	Median(Min-Max)	Median(Min-Max)	Median(Min-Max)		I→II	I→III	II→III
Necrosis	0 (0–1)	2 (1–3)	1 (0–2)	<0.001	<0.001	0.080	0.292
PMNL	2 (1–2)	2.5 (2–3)	3 (2–3)	0.002	0.019	0.006	1
MNL	1 (1–2)	2 (1–2)	1 (0–2)	0.066	NS.	NS.	NS.
Edema	1 (0–2)	1 (0–2)	1 (0–2)	0.668	NS.	NS.	NS.
Mucosal epithelium	2 (1–3)	3 (2–3)	2 (1–3)	0.010	0.016	1	0.047
Submucosal muscular layer	3 (2–3)	3 (2–3)	3 (2–3)	0.845	NS.	NS.	NS.
Granulation	2 (1–3)	2.5 (2–3)	2 (1–3)	0.219	NS.	NS.	NS.
Neoangio genesis	1.2 (1–2)	1.7 (1–2)	2.5 (2–3)	<0.001	0.207	<0.001	0.031
Fibroblast	2 (1–3)	3 (2–3)	3 (1–3)	0.027	0.027	0.017	1
Collagen deposition	1 (0–2)	1 (1–2)	1 (0–2)	0.736	NS	NS	NS

Kruskal–Wallis test (Monte Carlo), Post Hoc test: Dunn’s test; NS: not significant; Max: maximum; Min: minimum.

**Figure 2a:**
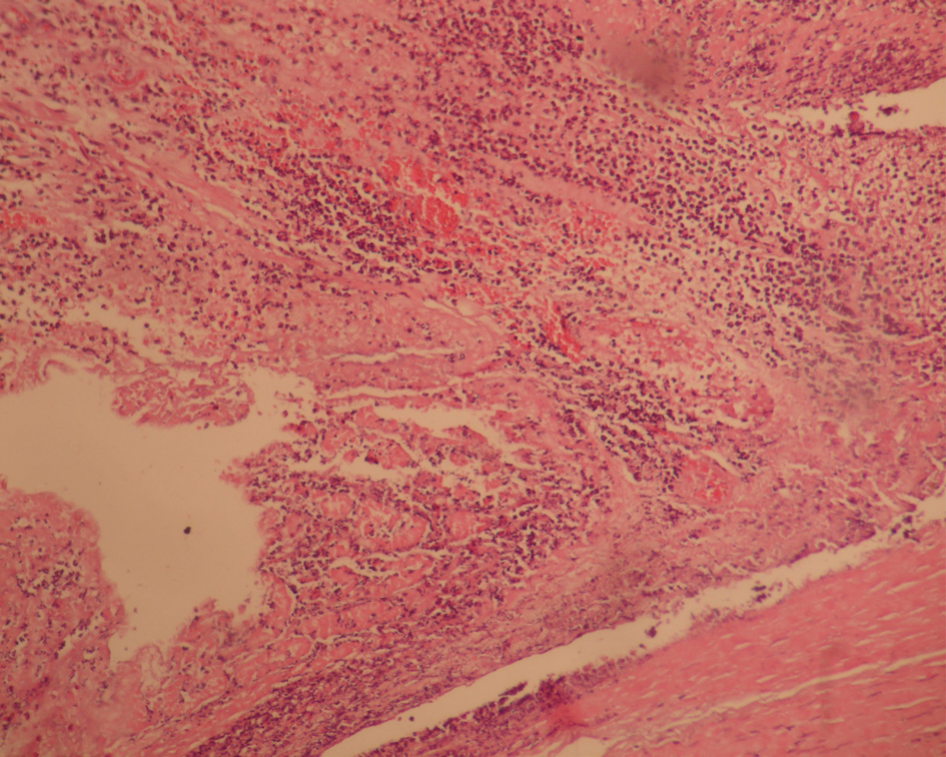
Microscopic appearance of the tissue on the 7th day in group III, lower necrosis (4x10, H&E).

**Figure 2b:**
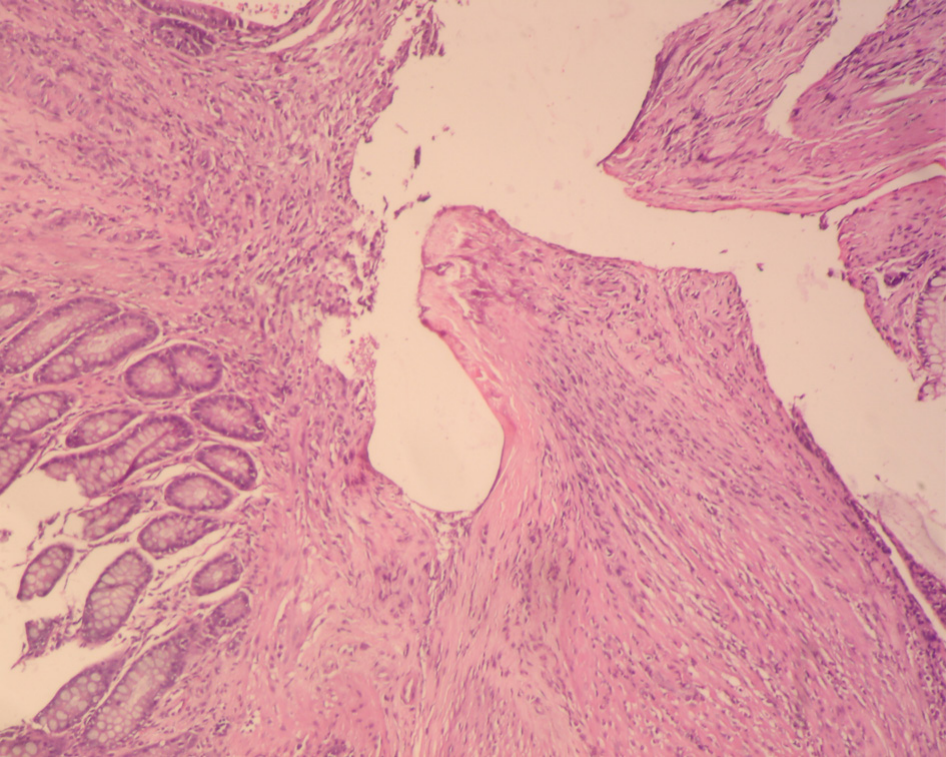
Microscopic appearance of the tissue on the 7thday in Group II, higher necrosis (4x10, H&E).

The PMNL infiltration rates were higher in groups II and III than those of group I on the 3rd and 7thdays (p = 0.027 and p = 0.008 for the 3rd day, respectively) (p = 0.019 and p = 0.006 for 7th day, respectively). However, the PMNL infiltration rates were similar in group II and III (p = 1.00) (Tables 4 and 5).

On day 3, the development rates of mucosal epithelium in group II and III were lower than group I (p < 0.010, p < 0.034, respectively). The rates of mucosal epithelium development in group II and III were similar (p = 1.00) (Table 4). On day 7, the development rates of mucosal epithelium in group II were lower than those of groups I and III (p = 0.016 and p = 0.047, respectively). There was no significant difference in the development rates of mucosal epithelium between groups I and III (p = 1) (Table 5).

On day 3, the rates of bridging between SBM and MS layers in groups II and III were lower than those of group I (p = 0.008 and p = 0.035, respectively). There was no significant difference between groups II and III in terms of bridging rates between SBM and MS layers (p = 1.00) (Table 4). On day 7, there was no difference among the groups in terms of bridging rates between SBM and MS layers (p = 0.845) (Table 5).

On day 3, the development rates of fibroblasts in all three groups were similar (p = 0.917) (Table 4). On day 7, the development rates of fibroblasts in groups II and III were higher than those of group I (p = 0.027 and p = 0.017, respectively). However, there was no difference between groups II and III (p = 1.00) (Table 5).

On day 3, the rates of neoangiogenesis development were similar among all groups (p = 0.642) (Table 5). On day 7, the rates of neoangiogenesis development were higher in group III compared to groups I and II (p < 0.001 and p = 0.031, respectively) (Figures 3a and 3b). However, there was no statistically significant difference between Group I and II in terms of neoangiogenesis development rate (p = 0.207) (Table 5).

**Figure 3a:**
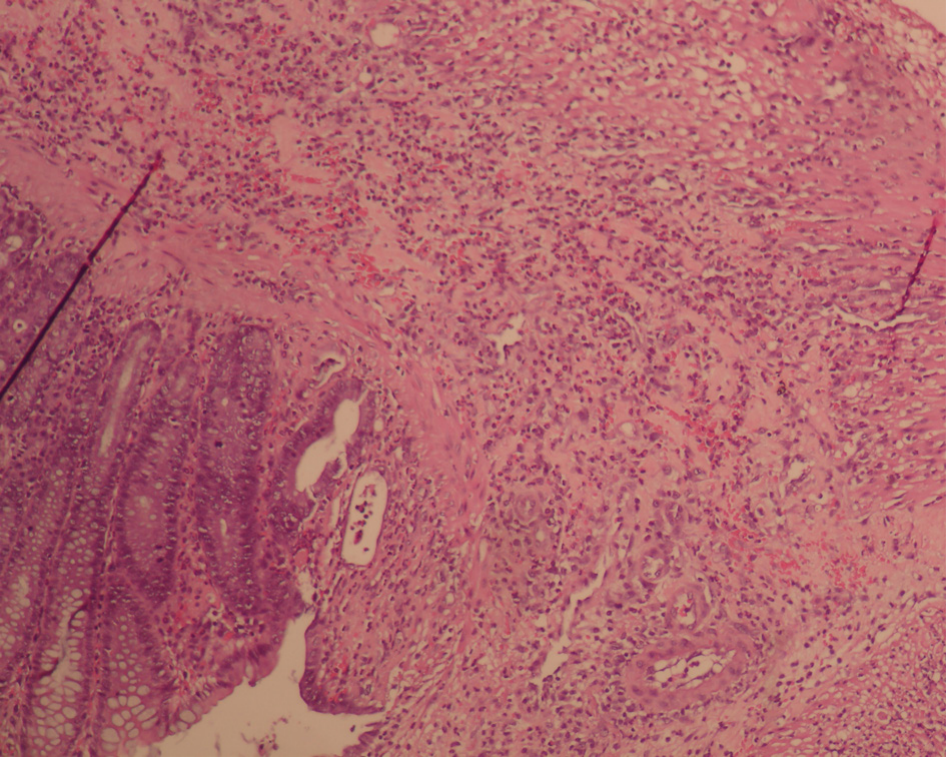
Microscopic appearance of the tissue on the 7th day in Group I, partial neoangiogenesis (10x10, H&E).

**Figure 3b:**
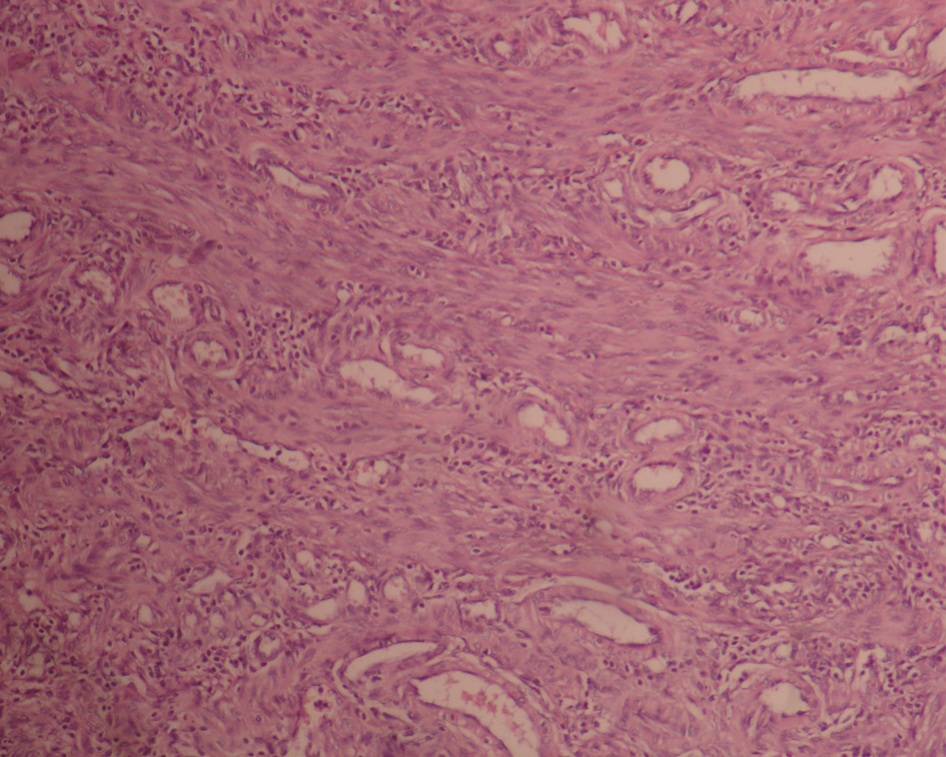
Microscopic appearance of the tissue on 7th day in Group III, marked neoangiogenesis (20x10, H&E).

On the 3rd and 7th days, there was no significant difference among the groups in terms of edema (p = 0.315 and p = 0.668, respectively), granulation (p = 0.471 and p = 0.219, respectively), and collagen deposition (p = 0.509 and p = 0.736, respectively).

## 4. Discussion

GI anastomosis healing is a systematic complex process of a certain time interval. Disruption in one of these stages via prolonged or excessive presence of cells in the anastomosis repair causes anastomotic failure by interfering with the entire healing process [20,21]. 

Ischemic damage deteriorates the healing process by causing proinflammatory cytokines to dominate the inflammatory phase of healing [22]. This study indicated the importance of systemic application of CORM-2 on colon anastomosis healing by decreasing cell destruction caused by ischemic damage and increasing collagen formation and neoangiogenesis. Cell destruction and elevated MDA levels occur as a result of the peroxidation of increased ROMs with unsaturated fats in cell membranes as the severity of inflammation increases in ischemic tissue [8,23,24].

In this study, PMNL infiltration and necrosis in the histopathological evaluation and MDA values in the biochemical evaluation were significantly higher in the ischemic groups (group II) on the 3rd and 7th day compared to the nonischemic group (group I). Irkorucu et al. [20] published that PMNL infiltration and necrosis were higher in the ischemia-induced left colon anastomosis than in the nonischemic group. These findings showed that the inflammatory process was more intense in ischemic colon anastomosis compared to nonischemic ones, and therefore oxidative stress might be dominant as well.

Experimental studies indicated that CORM-2 reduces cellular damage by reducing the duration and severity of oxidative stress, suppressing MPO activity, and inhibiting nuclear factor-κB (NF-κB), which increases the release of proinflammatory cytokines [25,26]. Adach et al. [26] stated that ROM (H_2_O_2_, H2O_2_/Fe) added to human plasma (in vitro) increased oxidative stress and addition of CORM-2 decreased lipid peroxidation. CORM-2 has been shown to reduce TNF-α (tumor necrosis factor), intercellular adhesion molecule 1 (ICAM-1), and E-selectin levels thus reducing PMNL infiltration, relieving tissue edema, and suppressing myeloperoxidase activity in small intestine ischemia-reperfusion injury model in rats [12]. 

CORM-2 has been shown to reduce inflammatory cell infiltration, tissue edema, and necrosis via the same mechanisms [13]. In this study, we found that the increased level of MDA in the tissue of the ischemia group (group II) have been decreased following the administration of CORM-2 (group III) on both the 3rd and 7th day. However, we also found that increased PMNL infiltration and edema in ischemic tissue did not decrease with the administration of CORM-2. These results show that CORM-2 suppresses cell damage and oxidative process, although it does not reduce PMNL infiltration caused by inflammation in ischemic anastomosis. ROMs cause endothelial damage, causing occlusion of vessels, and widening of necrosis [24]. Meng et al. [27] have investigated lipopolysaccharide-stimulated human umbilical vein endothelial culture and elaborated that CORM-2 exhibited an antithrombotic effect by inhibiting NF-κB and increasing thrombomodulin and endothelial protein C receptor activity that protect endothelial cells in the vascular endothelium. Magierowska et al. [28] reported that local necrosis of the gastric mucosa that the administration of CORM-2 resulted in increased tissue blood flow and significant prevention of cell necrosis via its antioxidative effect which was induced with 75% ethanol. Similarly, in this study, the increased histological necrosis on the 7th day of ischemia was reduced by 50% with the administration of CORM-2. Although this result was not statistically significant, the reduction of necrosis by half is an important finding. These findings suggest that CORM-2 causes a decrease in cell necrosis by preventing thrombosis of small vessels due to ischemia-related impaired blood flow by vasodilation.

Studies have shown that CORM-2 reduces apoptosis via caspase-3 inhibition and contributes to cell stability by increasing heat shock protein production via p38 kinase [29–31]. In this study, we observed that the antiapoptotic effect of CORM-2 might have contributed to the reduction of ischemia-induced necrosis.

In addition to its antioxidant function, glutathione has been shown to play a role in modulation of signal transduction, cell proliferation, and regulation of immune response [32,33]. In the study of Sawle et al. [34] the addition of CORM-2 to macrophage culture supplemented with lipopolysaccharide led to the increase of glutathione, which is a powerful antioxidant agent. Our results have been correlated with these outcomes and we found that administration of CORM-2 resulted in increase of the glutathione that have been decreased due to ischemia on the 7th day. These findings show that CORM-2 strengthens the intracellular antioxidant system while suppressing oxidative stress.

Neoangiogenesis is another physiological process that is critical in the wound healing [35]. Vascular endothelial growth factor (VEGF) is induced by hypoxia [36]. It can act as an angiogenic coactivator known as the hypoxia-inducible factor (HIF) [37]. HIF shows angiogenic effect by downregulating transcriptional cofactor PGC1β, which suppresses proangiogenic genes while inducing antiangiogenic genes [37]. Choi et al. [38] showed that addition of CORM-2 to the astrocyte cell culture stimulates angiogenesis by increasing VEGF synthesis and simultaneously increases the levels of heat shock protein HSP90, thereby enhancing the stability of HIF-1α and ensuring the continuation of the angiogenesis process. Similarly, Ahanger et al. [13] found that CORM-2 increased neovascularization in full-thickness skin wound model. In this study, we found that neoangiogenesis tended to increase in the ischemic group (group II) on the 7th day, compared to the nonischemic group (group I), but this difference was not statistically significant. We found that neoangiogenesis increased significantly with the administration of CORM-2 (group III) to the ischemic group compared to both the nonischemic (group I) and ischemic group (group II). These findings suggest that CORM-2 facilitates the cellular stability undergoing ischemia to maintain their viability and that CORM-2 creates a synergistic effect with angiogenesis.

Experimental studies showed that CORM-2 decreases intestinal epithelial damage due to inflammation and increases epithelial development in a full-thickness skin wound model [13,39]. In the experimental model of Uchiyama et al. [40] CORM-2 increased epithelial development by stimulating the synthesis of fibroblast growth factor-15 (FGF-15) from myofibroblasts. Similarly, in this study, we found that administration of CORM-2 increased the epithelial development that was diminished due to ischemia on the 7th day. These findings along with results from previous studies show that CORM-2 increases epithelial development in GI anastomosis.

In the proliferative period, smooth muscle cells and fibroblasts synthesize collagen, which forms the main structure of tissue strength. In this period, the decrease in collagen synthesis or increase in its degradation significantly increases the risk for anastomotic leaks [3]. Anastomotic strength can be assessed by intraluminal burst pressure measurement or by hydroxyproline, which can quantitatively indicate collagen deposition in tissue [3].

Similar to our outcomes, Irkorucu et al. [20] found that the hydroxyproline level decreased in the ischemic colon model. In this study, we found that hydroxyproline levels in the ischemic group (group II), which was not treated with CORM-2, was significantly lower than the nonischemic group (group I). These findings show that ischemia decreases collagen production in the GI anastomosis. In the experimental study with a full-thickness skin wound model, they found that the hydroxyproline level increased significantly in the group treated with CORM-2 compared to the group that was not [13]. Similarly, in our study, the hydroxyproline levels were higher in the ischemic group treated with CORM-2 (group III) compared to the untreated ischemic group (group II). These findings show that CORM-2 protects the cells involved in the synthesis of collagen, which is the main force of anastomosis, against oxidative stress in the inflammatory process and thus enables continuous functioning of these cells.

Previous studies have reported higher ABP in ischemic colon anastomosis compared to the nonischemic group [20]. In this study, the ABP was found to be significantly lower in the ischemic group not treated with CORM-2 (group II), compared to the nonischemic group (group I). We also elaborated that ABP tended to increase when CORM-2 was administered to the ischemic group (group III). However, this increase was not statistically significant. These findings show that CORM-2 provides balance in collagen synthesis and degradation and this balance may cause an increase in the physical strength of anastomosis.

## 5. Conclusion

In this study, we found that CORM-2 increases the level of antioxidant glutathione in the tissue, reduces the level of MDA, which is an indicator of cell destruction, and decreases the rate of necrosis histopathologically. In addition to these protective effects, we observed that CORM-2 increases tissue neovascularization, increases the level of HP in the tissue, and supports epithelial development. In the light of these findings, we think that the use of CORM-2 may have positive effects on GIS anastomosis healing. 

## Funding

## Informed consent

As this was an animal study, no informed consent has been obtained.
